# Semantic Representation and Emotional Awareness in Chinese Painting Viewing: Is There a Difference Between Landscape Painting and Figure Painting?

**DOI:** 10.3390/bs15060790

**Published:** 2025-06-09

**Authors:** Tinghu Kang, Ping Wang

**Affiliations:** School of Psychology, Northwest Normal University, Lanzhou 730070, China; kangyan313@126.com

**Keywords:** Chinese landscape painting, Chinese figure painting, semantic representation, emotional awareness

## Abstract

The artistic expression inherent in Chinese paintings serves as a conduit for the artists’ emotional and cognitive expression. However, current research lacks consensus regarding the distinct psychological mechanisms underlying the appreciation of Chinese painting genres (landscape vs. figure paintings). This study—employing a vocabulary generation task and the Implicit Association Test (IAT) to compare semantic representation and emotional awareness during participants’ viewing these two types of paintings—aims to elucidate potential disparities in aesthetic processing. In Experiment 1, although both types of paintings produced an abundance of content words, figure paintings elicited a greater number of emotional association words than landscape paintings. Meanwhile, Experiment 2 demonstrated faster response times for an incompatible joint task versus a compatible joint task. These findings collectively suggest that the aesthetic of paintings may engage automatic processes, with the effects on semantic representation and emotional awareness appearing to be independent of the type of paintings. The predominance of content processing over emotional response may be attributed to the temporal characteristics of emotional arousal.

## 1. Introduction

The aesthetic experience is a cognitive process accompanied by continuously upgrading affective states that vice versa are appraised, resulting in an (aesthetic) emotion (Leder et al., 2004). This multifaceted phenomenon, integrating perception, cognition, and emotion, remains central to art research. The objective properties of artworks, such as symmetry, complexity, contrast, and typicality (Jacobsen et al., 2006; Imamoglu, 2000; Halberstadt & Rhodes, 2003), as well as subjective factors, including expertise, implicit memory and imagination, familiarity, and processing fluency (Seeley, 2006; Belke et al., 2010), operating through distinct mechanisms, also contribute to the aesthetic appraisal. This intricate relationship between art and viewers has been a focal point of psychological research, revealing that different types of paintings elicit distinct aesthetic evaluations and even activate different brain regions, which has been confirmed by psychological research on the viewing of Eastern and Western paintings (Chua et al., 2005; Lu et al., 2015). Recent studies have highlighted the differences in cognitive processing between representational and abstract art forms, with a general preference for figurative art over abstract art being documented (Feist & Brady, 2004; Suhaili et al., 2024). This preference is thought to be rooted in the distinct cognitive mechanisms underlying the perception of these art forms, which remains an active research frontier.

Chinese painting is creating by the predominant use of ink-wash, complemented by mineral pigments, employing brushes as painting tools and paper as the compositional medium. Rooted in Daoist philosophy, it operationalizes two core philosophical principles: Tianren Heyi (天人合一, unity between heaven and man) and Xushi Xiangsheng (虚实相生, virtual–real fusion), features that distinguish it from Western painting traditions. The development of Chinese painting has a long history, originating in the pottery paintings and rock paintings of primitive society. At that time, the paintings used simple lines to depict the prominent characteristics of things so as to express the good wishes and beliefs of people. Technical sophistication progressed through dynastic eras; the techniques of painting have also been greatly improved. In the period of Wei and Jin, Southern and Northern Dynasties, there appeared what is now called Chinese painting. Most of the paintings during this period were dominated by figure paintings, and most of the styles of paintings were pursued freely and easily. In the Sui and Tang Dynasties, landscape painting emerged as an autonomous genre and outlined the state of mountains and rivers with meticulous lines to express the painter’s emotion in pursuing an interest in landscapes (Jiang, 2008; Q. Yang, 2022). Of each type of Chinese painting with its distinct thematic content and emotional expression, landscape painting mainly depicts the natural scenery of mountains and rivers, focusing on the harmony between nature and the self. The content of figure painting is based on the depiction of people, highlighting human emotions and social interactions (Jiang, 2008; Q. Yang, 2022).

Chinese painting fundamentally realizes the dialectical unity of affective resonance and objective reality, the essence is rooted in the integration of emotions with objects, a core principle that has consistently defined Chinese painting throughout its history. The creation of Chinese painting represents a fusion of objects and emotions, and by blending these two elements, the artistic realm of the artwork is enhanced. As one of the fundamental concepts of artistic dialectics, artistic conception consists of two dimensions: “Emotional Intentionality (Yi 意)”, which refers to the unity of emotion and reason, belonging to the subjective realm; and “Formal Manifestation (Jing 境)”, which refers to the unity of form and spirit, belonging to the objective realm. Through their continuous interaction and mutual integration, form and spirit, and emotion and reason interpenetrate and constrain each other, ultimately forming “artistic conception (Yijing)” (S. Yang, 2011). Through this integrative process, artworks transcend representation to achieve scene–emotional permeation (scene embodies emotion, and emotion permeates the scene), where emotional states become materially encoded in brushstrokes and compositional elements. Crucially, the cognitive reception of these emotional states may vary significantly between landscape paintings and figure paintings due to their divergent themes: while landscape paintings invite the contemplation of transcendent natural orders, figure paintings emphasize interpersonal dynamics and social realities. Chinese landscape painting and figure painting feature huge differences in both the painting content and the emotion conveyed by the painting, which may also lead to varied understandings and aesthetic experiences of the different types of paintings by the viewers. The current research on art cognition has predominantly focused on Western art, neglecting different cultural traditions’ influence on cognitive processing. This study aims to examine the cognitive mechanisms underlying the viewing of Chinese landscape paintings and figure paintings.

Previous research on the cognitive processing of art has provided nuanced perspectives on aesthetics, offering detailed insights from both cognitive and emotional dimensions. Empirical evidence suggests that the aesthetic appreciation of art is based on their cognitive processing of the artwork, and that the content of the artwork, contextual knowledge, and viewers’ prior professional background and relevant experience all determine their aesthetic experience (Leder et al., 2004). However, these studies often neglect the emotional aspects of aesthetic processing. Another part of the research argues that aesthetics is emotion-based, that they are intuitive reflections of artworks, and that affective judgments exist before perceptual and cognitive operations take effect (Zajonc, 1980). What kind of psychological response cognition and emotion will have on aesthetics during the appreciation of artworks remains a subject of ongoing scholarly debate. In a groundbreaking eye-tracking study, Kang et al. (2023) systematically compared the visual exploration patterns between Chinese landscape paintings containing figures and those without figure elements. The findings indicated that the aesthetic way was different, and the aesthetic experience results were also different even within the same genre, because of the different information they contain. Building on this foundation, the current investigation using a vocabulary generation task (Roghani & Milton, 2017) and Implicit Association Test (IAT) to deconstruct the cognitive–affective interplay in the appreciation of Chinese landscape paintings and figure paintings.

To establish the theoretical framework, this study employs the information processing model of aesthetic experience proposed by Leder et al. (2004), which integrates the cognition and emotion of the aesthetic experience and helps to understand the mechanisms involved in cognitive and affective processes in the aesthetic process of visual art. This model posits that the aesthetic experience is a multistage process, involving aesthetic input, mental processing, and aesthetic output. Leder and Nadal (2014) subsequently refined this theoretical model through empirical studies, demonstrating that emotional states are no longer simply the result of a simple output but are a temporary overlap of the time course of the prior cognitive processing stages. According to this theory, the art perception involves the dynamic interaction between sensory processing and cognitive–emotional modulation. For example, the aesthetic appreciation of representational art may rely more on semantic and memory-related processes, while abstract art may engage perceptual and emotional systems more directly (Bimler et al., 2019; Gernot et al., 2018). In addition, from the perspective of automatic evaluations, this theory also finds that the aesthetic experience may be unconscious, immediate, and purely stimulus-driven, together with the findings showing that aesthetic evaluation sometimes forms rather quickly, suggesting that automatic processes of aesthetic judgment might take place even before the higher cognitive processing of meaning takes effect (Leder et al., 2004; Leder & Nadal, 2014; Locher et al., 2007). The finding of Pavlovic and Markovic (2012) further indicate that aesthetic evaluations derive partially from automatic hedonic responses to artistic stimuli. Aesthetic judgments can be formed immediately in the form of immediate emotional responses. In the context of our study, this theory framework provides a robust foundation for investigating cognitive processes in the perception of Chinese landscape paintings and figure paintings. Through comparative analysis of these two distinct styles, this study aims to explore how different artistic forms influence the cognitive and emotional processes involved in art appreciation.

The primary objective of this study is to investigate the cognitive processes underlying the perception of Chinese landscape paintings and figure paintings. According to Piaget’s theory of cognitive development, the cognitive level of high school students has already developed to the formal arithmetic stage, and the thinking ability has gradually developed and matured, with increasingly rich imaginative ability and abundant emotions. Consequently, high school students have obviously demonstrated their ability to judge independently and appreciate beauty in the field of aesthetic activities, and their aesthetic psychological structure has also developed rapidly. They have a profound sense of beauty and great plasticity for beautiful things, and they have a unique aesthetic psychological structure and aesthetic psychological characteristics. In addition, the Ministry of Education of the People’s Republic of China has decided to comprehensively implement the school aesthetic education action to enhance the aesthetic quality of students. As an important stage of aesthetic education, investigation into the aesthetic cognition patterns of high school students can provide critical empirical support for optimizing pedagogical strategies in arts education. Therefore, this study selected high school students as participants to further understand their aesthetic characteristics and cognitive processing differences regarding different art forms, such as landscape painting and figure painting. This study addresses the following research question: Do Chinese landscape paintings and figure paintings elicit distinct semantic representation and emotional awareness responses? Based on the literature on art cognition and the unique characteristics of these two painting styles, this study hypothesizes that the aesthetic experience in Chinese painting is not merely a passive reception of visual stimuli, but an active cognitive and emotional engagement; the viewing of both types of paintings may involve an automatic process and mainly involves the acquisition of painting content, but figure paintings evoke more emotion than landscape paintings.

## 2. Experiment 1

### 2.1. Participants

A total of 98 general high school students, 51 males and 47 females, with a mean age of 15.18 ± 0.54 years, were selected. The required sample size was calculated using G*power 3.1 software (Faul et al., 2009) with an effect size *f* = 0.25, significance level size *α* = 0.05, and power size 1 − *β* = 0.8. Thus, this experiment met the minimum sample size requirement. Through interviews, it was learned that all participants had only received training in drawing techniques, such as sketching within the educational system, with an absence of extracurricular art training or specialized art education, and all participants were right-handed, with normal visual acuity or corrected visual acuity and no color blindness or color weakness. Parents had been informed of the whole experimental process, both the parents and the participants filled out an informed consent form before the experiment began, and they were paid at the end of the experiment. The study was conducted in accordance with the Declaration of Helsinki, and this study was approved by the Ethics Committee of the Northwest Normal University, China, Approval Number: 2024276, dated: 27 May 2024, for studies involving humans.

### 2.2. Materials

**Figure 1:**
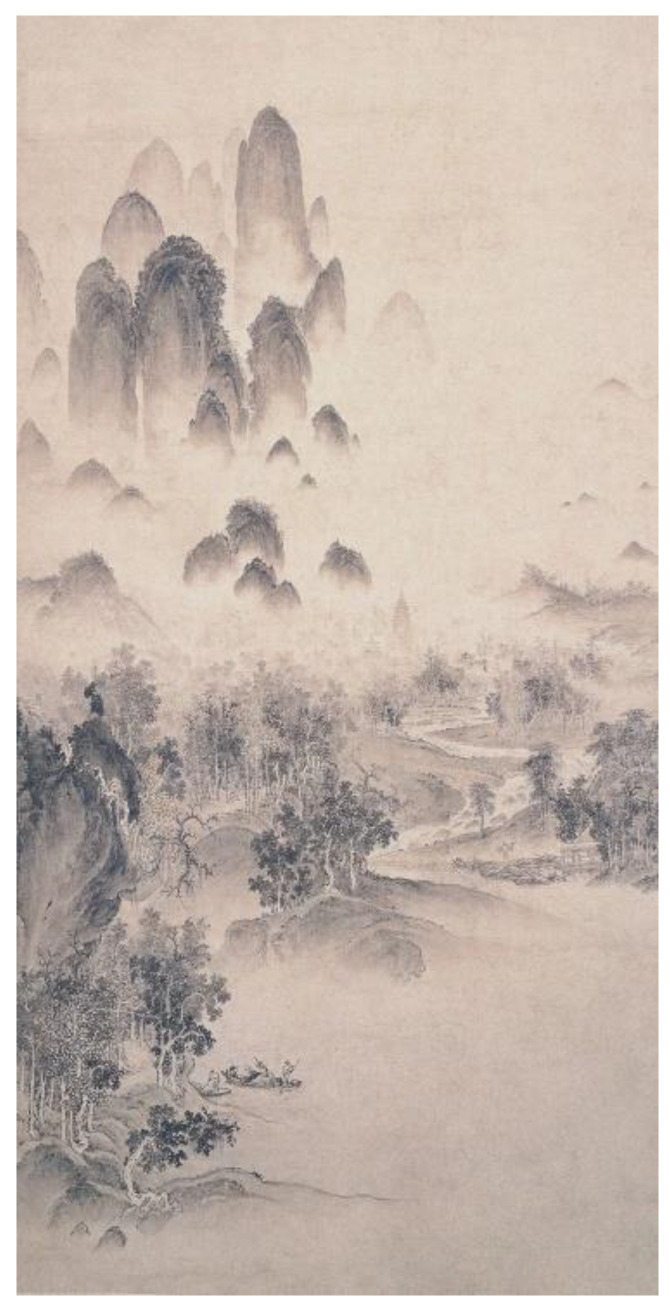
A sample of Chinese landscape painting. This painting was painted by Zhao Ji, a painter of the Northern Song Dynasty. The painting is titled *Autumn Scenery of streams and Mountains*.

**Figure 2:**
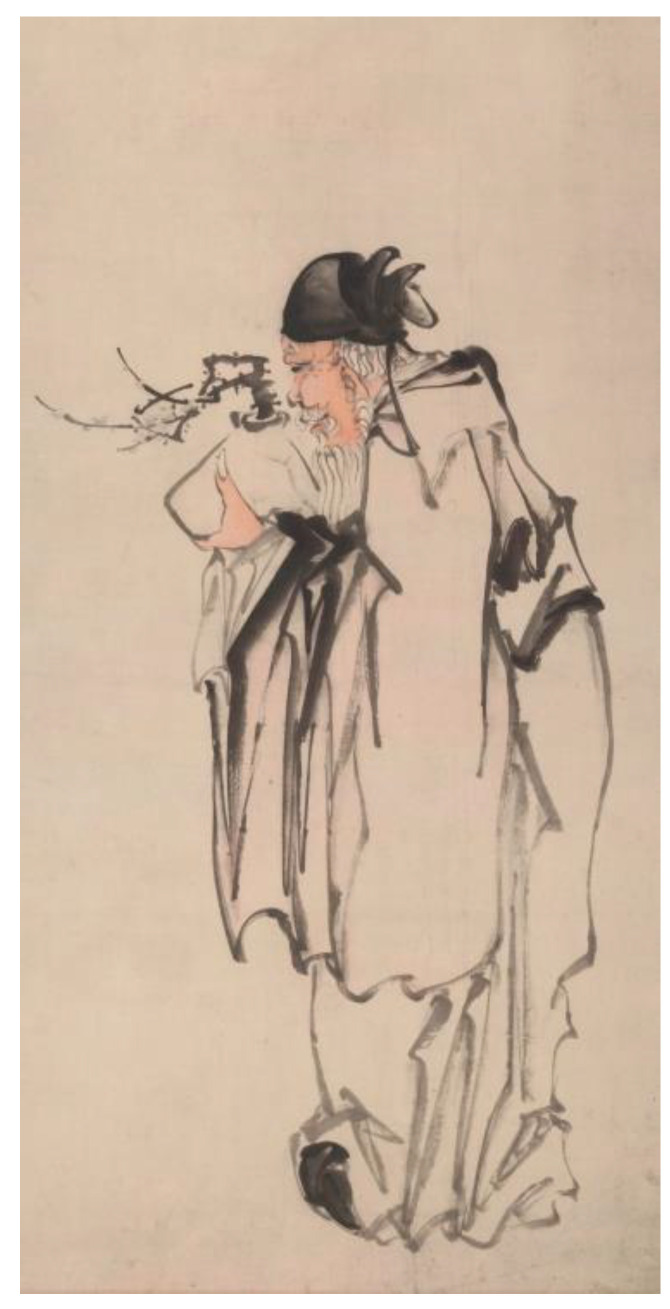
A sample of Chinese figure painting. This painting was painted by Huang Shen, a painter of the Qing Dynasty. The painting is titled *Holding Plum Blossoms*.

Vertical scroll painting, a format characterized by traditional Chinese cultural features, remains uncommon in Western artistic traditions due to its distinctive aspect ratio where vertical dimension substantially exceeds horizontal width. In this study, 20 Chinese ink landscape paintings and figure paintings each were selected, excluding brushwork. All artworks were standardized to 1604 × 3200 pixels through Photoshop software during which extraneous elements (e.g., artist seals, marginal inscriptions) unrelated to pictorial content were digitally removed. The professional participants (doctoral or master’s degree students in Chinese painting, 3 males and 7 females, with an average of 7.2 ± 2.74 years of study in Chinese painting) were required to classify each painting according to its theme content (landscape painting or figure painting) based on their professional knowledge of Chinese painting classification and to rate the degree of affiliation by 5 points, and finally 10 landscape paintings (M = 4.87, SD = 0.05) and 10 figure paintings (M = 4.8, SD = 0.11), each with the highest average rating, were selected as experimental materials. Figure 1 displays a representative ink-wash Chinese landscape painting, while Figure 2 exemplifies a classical Chinese figure painting.

### 2.3. Instrument

Lenovo desktop computers in a high school microcomputer room were used, with a 19-inch LCD display, a desktop resolution of 1920 × 1080, and a refresh frequency of 59.934 Hz. The computers were all installed with Windows 10, and the paintings are presented randomly by the master computer in the form of images.

### 2.4. Procedure

This experiment employed a vocabulary production task (Huang, 2012) in which participants were not provided with any information about the paintings and were asked to write down three words that come to mind when they viewed each Chinese painting. These words should be able to describe the painting, such as the ideas generated by the participant after viewing the painting, the content of the painting, or whether the participant feels the emotion conveyed by the painting, and so on; there is no limit to the types. However, participants were asked to try not to write general words such as “nice”, “like”, “beautiful”, etc. The range of speech and content of the generated keywords shall not be restricted.

Through a simple analysis of the recovered vocabulary, it was found that the vocabulary generated by the participants could be divided into three main categories: content words, emotional association words, and other words, which was basically consistent with the findings of Huang (2012), so this experiment also used these criteria to classify the words. Content words refer to the participants’ descriptions of the objective things depicted in the paintings and their attributes, e.g., mountain, suona, turbulent, old, etc. Emotional association words refer to participants’ descriptions of the thoughts and emotions expressed in the paintings and the content that did not appear in the painting when they were viewing the painting, e.g., high, lonely, decayed, etc. Other words refer to the descriptive words other than the first two types of words, which generally include the painter’s painting skills and other aspects, such as fluent, light, etc. Therefore, there will be no further analysis of such words in this study.

The experiment was a one-way experimental design in which the independent variable was the type of painting (landscape paintings/figure paintings), and the dependent variable was the number of different types of words generated by the participants.

### 2.5. Results

All of the words generated by the participants were classified by three graduate students from the College of Chinese Language and Literature at the same time, and the classified words were rated again by 15 graduate students (none of them participated in the previous classification session) from the College of Chinese Language and Literature after the classification. The frequency of occurrence of each vocabulary category in each painting was used as an indicator to compare whether participants had differences in aesthetics between the two different types of Chinese paintings. For landscape paintings, participants generated 1407 content words and 292 emotional association words. For figure paintings, participants produced 979 content words and 706 emotional association words. The results showed that the painting type that generated the most content words was landscape painting, and the painting type that generated the most emotional association words was figure painting, for which the number of content words generated was still greater than the number of emotional association words. The results of the average number of different types of words generated by different types of Chinese painting are shown in Table 1.

To compare the frequency with which participants generated different types of words for two different types of Chinese paintings, the Chi-square test was used as indicated in the Table 2.

Using the Chi-square test, the results show that Pearson *χ*^2^ = 248.46, *df* = 1, *p* < 0.05, indicating that the row variable painting type and the column variable word type are not independently related to each other and thus have some correlational effects; in other words, there are significant differences in the types of words generated between different types of Chinese paintings.

**Figure 3:**
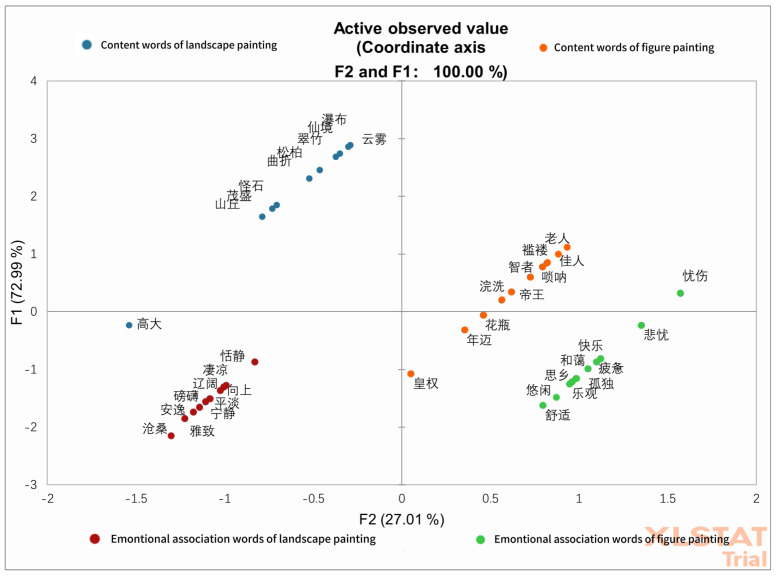
Perceptual map of the different concepts of types of Chinese paintings in the two components of the principal correspondence analysis. Content words of landscape painting: 瀑布 (waterfalls), 仙境 (fairyland), 云雾 (clouds and mist), 翠竹 (green bamboo), 松柏 (pine trees), 曲折 (winding paths), 怪石 (strange rocks), 茂盛 (lush vegetation), 山丘 (hills), 高大 (tall trees); emotional association words of landscape painting: 恬静 (tranquil), 凄凉 (desolate), 辽阔(vast), 向上 (uplifting), 平淡 (plain), 磅礴 (majestic), 宁静 (peaceful), 安逸 (comfortable), 雅致 (elegant), 沧桑 (weathered); content words of figure painting: 老人 (old man), 佳人 (beautiful woman), 褴褛 (ragged clothes), 唢呐 (suona), 智者 (wise man), 帝王 (emperor), 浣洗 (washing clothes), 花瓶 (vase), 年迈 (elderly), 皇权 (imperial power); emotional association words of figure painting: 忧伤 (sadness), 悲忧 (grief), 快乐 (happiness), 疲惫 (fatigue), 和蔼 (affability), 孤独 (loneliness), 思乡 (homesickness), 乐观 (optimism), 悠闲 (leisure), 舒适 (comfort).

The vocabulary generation data were analyzed qualitatively by grouping the terms with similar meanings into different categories; the terms mentioned by at least 10% of the participants were considered for quantitative analysis to avoid losing a large amount of information (de Andrade et al., 2016). All data analyses were conducted using XLStat© software (version 2019, Lumivero, Denver, America), and results were considered significantly different at a significance level of *p* ≤ 0.05. A simple Principal Correspondence Analysis (PCA) was conducted in order to obtain the perceptual map of the different types of Chinese paintings and vocabularies generated by individuals while viewing (Figure 3). A contingency table with bi-dimensional representations was obtained, where rows corresponded to the different types of Chinese paintings, and columns represented the generated vocabularies. The first two dimensions of the PCA showed 100% of the data variability, representing 72.99% and 27.01% of the variance, respectively. The perceptual map showed differences among stimuli, observing two groups of stimuli (Figure 3).

## 3. Experiment 2

### 3.1. Participants

A total of 50 high school students from general high schools were selected, 25 males and 25 females, with a mean age of 15.22 ± 0.47 years. The required sample size was calculated using G*power 3.1 software (Faul et al., 2009) with an effect size *f* = 0.25, significance level size *α* = 0.05, and power size 1 − *β* = 0.9. Therefore, this experiment met the minimum sample size requirement. All participants had not participated in Experiment 1 or similar experiments, and the other characteristics were consistent with Experiment 1. The study was conducted in accordance with the Declaration of Helsinki, and this study was approved by the Ethics Committee of the Northwest Normal University, China, Approval Number: 2024276, dated: 27 May 2024, for studies involving humans.

### 3.2. Materials

A total of 10 paintings each of landscape paintings and figure paintings were selected from Experiment 1.

The 20 words in the IAT were divided into objective and subjective words. The material was selected from Experiment 1 describing 20 content words and 20 emotional association words each with the highest frequency by participants, for a total of 40 words. A group of 27 graduate students (8 male, 19 female; none of them participated in the previous commentary session) of the College of Chinese Language and Literature rated the degree of their affiliation by 5 points and finally selected the 10 highest-scoring content words (*M* = 3.72, *SD* = 0.23) as the objective word and the 10 highest-scoring emotional association words (*M* = 3.91, *SD* = 0.29) as the subjective words for this study. The objective words (from content words of Experiment 1) were finally determined as follows: hill, megaphone, cloud, pine and cypress, waterfall, old man, strange rock, ragged, lush, and fairyland (山丘, 唢呐, 云雾, 松柏, 瀑布, 老人, 怪石, 褴褛, 茂盛, 仙境); the subjective words (from the emotional association words of Experiment 1) were tired, worried, lonely, sad, happy, desolate, optimistic, homesick, at ease, and serene (疲惫, 忧伤, 孤独, 悲忧, 快乐, 凄凉, 乐观, 思乡, 安逸, 平淡).

### 3.3. Procedure

In the study of automated processing, the Implicit Association Test (IAT) is the classic research paradigm. It has been shown that automated processing between paintings and attributions can be responded to by implicit association tests (Snagowski et al., 2015). Pavlovic and Markovic (2012) employed the IAT with the aim of examining the nature of automatic aesthetic judgment in figural art and abstract art. This experiment used a 2 (type of painting: landscape paintings/figure paintings) × 2 (attribute word: objective word/subjective word) within-group experimental design, with the independent variables being the type of painting (landscape paintings/figure paintings) and attribute word type (objective word/subjective word), and the dependent variable indicator being the response time of the participants under different task conditions.

The IAT procedure assesses implicit self-esteem by using 10 landscape paintings and 10 figure paintings selected from the pre-material ratings as items of the painting type, and 10 subjective and 10 objective words as items of the vocabulary type. The order in which each painting and each vocabulary word appeared was balanced.

To ensure the participants’ understanding of the IAT procedure, participants first completed a short practice experiment in which the material used was unrelated to the formal experiment. After the practice experiment, the IAT procedure consisted of a total of seven categorized blocks (see Table 3), with a practice block (block 1, 2, 3, 5, and 6) of 10 trials and a data collection block (block 4 and 7) of 20 trials. In the experimental procedure, the corresponding keystroke instruction was presented before the start of each block. The participants were required to respond to different types of stimuli according to the instructions. The experiment required the participants to complete the keystrokes as quickly as possible under the premise of ensuring the accuracy of the responses. The specific stimulus–response mapping relationships and key requirements are shown in Table 3. Each stimulus item was displayed until the correct response was made. The next stimulus item then followed after a 150 ms intertribal interval. The computer recorded the elapsed time between the start of each stimulus word’s presentation and the occurrence of the correct keyboard response. All blocks were practice blocks except for the two critical blocks (block 4 and 7) from which data were used to calculate the IAT effect. Items in each category were randomly selected without replacement so that all items were used once before any item was reused.

### 3.4. Results

Experimental data were selected for the reaction times of the compatible joint task and the incompatible joint task, i.e., phases 3 and 4 and phases 6 and 7. Convert reaction times were below 300 ms to 300 ms and above 3000 ms to 3000 ms (Cai, 2003). Next, all the raw response time data were log-transformed, and then the mean reaction time was calculated separately for the compatible and incompatible groups (part IV and part VII in this example). Finally, the mean response time of the incompatible group was subtracted from the mean reaction time of the compatible group so that the resulting score was the extent to which the participant associated the content word with the landscape painting relative to the emotional association word, i.e., the effect value of IAT and its logarithmic value. The specific data are shown in Table 4.

The results of the paired samples t-test indicated that there was a correlation between the paired samples and that the participants did not differ significantly when responding to the compatible joint task and the incompatible joint task, *t*(49) = 0.72, *p* > 0.05, Cohen’s *d* = 0.03. The difference in the value of the IAT effect pairs was significant, *t*(49) = 52.25, *p* < 0.05, Cohen’s *d* = 3.35.

Using the experimental principle of the IAT, individuals respond to a painting and a vocabulary faster and more easily if the aesthetic processing adopted by the individual to appreciate the painting and perceive the vocabulary is consistent in some properties, and vice versa. In the present experiment, we found that when participants categorized the paintings and vocabulary by painting type and vocabulary type, participants performed more response times when performing the compatible association task than when performing the incompatible joint task, and the experimental results do not further confirm that landscape paintings are more strongly associated with the content vocabulary (objective criterion) and figure paintings are more strongly associated with the emotional joint class vocabulary (subjective criterion).

## 4. Discussion

### 4.1. Are There Differences Between Semantic Representation and Emotional Awareness in Chinese Painting Viewing?

As one of the traditional Chinese cultural arts, the value and significance of Chinese painting are intrinsically evident. Experiment 1 employed the vocabulary generation task to investigate the semantic representation and emotional awareness of individuals in the process of viewing Chinese paintings. The results revealed that regardless of the type of painting, content words were produced the most, but in comparison, figure paintings produced more emotional association words than landscape paintings.

The landscape paintings and figure paintings selected in this study are painters’ depictions of real-life patterns and natural scenery, which undergo subsequent artistic processing, refinement, and abstraction while retaining the fundamental characteristics of their subjects so that the painting still retains the basic characteristics of objective things and has a certain degree of realism. The content depicted is part of Chinese culture and is easily recognizable and understandable to Chinese people. When analyzed from the perspective of semantic representation, the content word is the recognition of objective things in the painting, which belongs to object recognition at the perceptual level and is at a relatively primary stage of processing. As the painter captures the basic characteristics of things in artistic creation, when an individual appreciates a Chinese painting in a short period of time, the first thing the individual recognizes is the content of the painting, which is the objective thing depicted by the painter. The information available to the individual is the perception of the content of the painting, which leads to the highest content words produced when viewing a Chinese painting.

According to the information processing model of aesthetic experience proposed by Leder et al. (2004), the interplay between top-down and bottom-up processing culminates in the aesthetic experience. The emotional state results from continuous evaluation and its interaction with cognitive processes, with emotional responses evolving dynamically as the individual progresses from perceptual analyses to evaluation (Leder et al., 2004). For example, initial perceptual analyses with a painting may elicit emotional responses (e.g., surprise, curiosity), and these emotions will be amplified or modulated with further processing of the painting (e.g., understanding of the style and content of the painting). In this study, the relatively few emotional association words individuals express when viewing paintings may be due to the early stages of aesthetic perception. Individuals need to experience a certain amount of time for the emotional output of art, and a short viewing may not allow individuals to experience the deeper emotions expressed in Chinese paintings, thus constraining emotional association word output. In addition, for abstract concepts that lack perceptual features, there is no clear counterpart in the objective world (e.g., “boredom”, “delight”), so individuals cannot acquire such abstract concepts directly through any visual, tactile, auditory, and other sensory channels. It has been shown that words representing concrete concepts (e.g., “apple”) are processed faster and more accurately than words representing abstract concepts (e.g., “ideal”) in tasks such as sentence judgment, semantic classification, and lexical judgment (West & Holcomb, 2000). This disparity in processing speed and accuracy extends to tasks involving recognition, free and cued recall, and paired association learning, where concrete words are favored over abstract ones. As a result, emotional association words, as words representing abstract concepts, are acquired and processed more slowly than content words representing concrete concepts, which is reflected in the fact that fewer words are generated than content words.

During visual processing, the figure painting has a much higher number of emotional association words than the landscape painting, a difference that likely stems from their distinct content characteristics. Lazarus (1991) proposed that aesthetic experiences can evoke any kind of emotion, such as gratitude and empathy; there is no single and unique aesthetic emotion. Since the subject of the figure painting is the figure, when viewing the painting, the individual can more easily appreciate the thoughts and emotions that the artist has put into the painting through the perception of the figure’s form and the association of the figure with the surrounding scene. When individuals view artwork, they can empathize with the artwork through embodied experience (Freedberg & Gallese, 2007). When viewing paintings, viewers project affective states onto depicted figures, constructing a subjective experience of the content of the painting and “empathizing” with the figures in the paintings. In addition, faces play an important role as stimuli in physiological and social stimulation, which may have an attentional advantage over other objects, and can be processed efficiently and quickly to attract the attention of individuals (Crouzet et al., 2010; Ro et al., 2001; Langton et al., 2008). When viewing the figure painting, if the attention is prioritized on the faces in the painting, and facial expressions of faces are recognized and understood, it is easier to produce emotional association words.

### 4.2. Is the Automatic Processing of Semantic Representation and Emotional Awareness Affected by the Type of Painting?

Based on Experiment 1, the IAT was employed to measure the automatic aesthetic evaluation of visual stimuli. The results indicated that individuals responded longer to performing the compatible joint task than to performing the incompatible joint task, but this result was not statistically significant. Combining the results of Experiment 1 and Experiment 2, there was no significant difference in semantic representation and emotional awareness of landscape painting and figure painting.

In the study, the reaction time for the compatible joint task was longer than the response time for the incompatible joint task, which is inconsistent with the hypothesis of IAT. On the one hand, the reason for this result may be related to the content of the paintings. From the perspective of visual processing, the physical characteristics of aesthetic objects may affect people’s cognitive processing and aesthetic experience (Villani et al., 2015). In addition to the obvious mountains and water, there may also be details such as people and animals in landscape paintings, which may increase response time when individuals further process the details in the paintings. On the other hand, it may be related to the theme of the painting, e.g., the content of the figure painting is developed around the figure, and individuals can get a glimpse of the figure while viewing, but they need to further empathize with the emotions implied by the figures in the paintings, and the process of empathizing may lead to an increase in response time.

Indeed, Leder et al. (2004) proposed an information processing model of aesthetic experience, which describes a series of processing stages during aesthetic engagement. One of these stages is described as “implicit memory integration”, referring to automatic cognitive processes such as familiarity and typicality detection when evaluating artistic stimuli. Supporting this framework, Locher et al. (2007) demonstrated that a brief glimpse of a digitized artwork on a computer screen for 100 ms is enough for the viewer to discover a great deal of information about it, not only structural features, such as shapes and colors, but also more conceptual aspects, such as the elements depicted in the scene. Furthermore, individuals employ distinct cognitive processing methods when appreciating landscape painting and figure painting, and their aesthetic experience and feelings are also different (Davide et al., 2012).

The individuals tend to pay more attention to the emotion contained in the figure painting, and process figure painting more from the perspective of emotion. While individuals are typically expected to evaluate Chinese paintings aesthetically during brief viewing periods, participants in this study exhibited no preference for specific categories of Chinese paintings in aesthetic processing. This is probably because regardless of the kind of Chinese painting, the individuals lacking aesthetic expertise primarily focus on identifying pictorial content through semantic representation during the initial stage of aesthetic processing regardless of the specific style of Chinese painting being evaluated. At this stage, they do not differentiate between artistic categories, as all Chinese paintings are perceived generically as “artworks” rather than being assessed for their stylistic value. To untrained viewers, these Chinese paintings are uniformly categorized as “paintings” without stylistic distinction.

## 5. Limitations and Future Direction

This study is not without caveats and other issues for future research. First, the sample included only high school students, which may somewhat limit the generality of the findings. The age range (15–18 years old) and stage of cognitive development (formal arithmetic stage) of high school students may make their aesthetic experience different from those of other age groups (e.g., adults or children). In addition, the lack of professional art training in high school students may make their cognitive processing and emotional response to artworks different from that of art professionals. Future studies could be extended to participants of different age groups and backgrounds to verify the generality of the findings of this study.

Second, this study excluded themes with high emotional intensity (e.g., religious or offensive content) when selecting paintings to ensure homogeneity and controllability of the experimental material. However, this choice may have limited the breadth of the aesthetic experience captured by the study, as artworks with a high emotional intensity may have elicited stronger or different cognitive and emotional responses. Future studies could include more types of artworks to explore the effects of different emotional intensities on aesthetic experience.

Thirdly, the behavioral measures employed in this study may not have been able to fully capture the participants’ aesthetic experiences, while physiological metrics (e.g., eye movements or brain waves) may have provided more objective data. Future research could combine multiple measures to more fully assess the complexity of the aesthetic experience.

Finally, laboratory control conditions, while necessary to control additional variables, may not fully reflect the complexity and variability of real-world scenarios, inevitably sacrificing ecological validity. The specific lighting, seating arrangements, and absence of external distractions in a laboratory differ significantly from the diverse contexts in which art is typically encountered, such as galleries, museums, or public spaces. This artificial setting may alter the participants’ aesthetic experiences and cognitive processing in ways that are not representative of real-world art viewing. Future research could consider conducting studies in more natural and varied settings to better understand how different environmental factors influence aesthetic experiences and to enhance the ecological validity of the findings. Despite these limitations, this study provides preliminary evidence for understanding the differences in cognitive processing of different art forms among high school students and points the way for future research.

## 6. Conclusions

This study investigates the characteristics of individuals’ aesthetic processing when viewing Chinese landscape painting and figure painting through behavioral experiments. Under the short-time viewing conditions, there is no significant difference in the individual’s aesthetic processing mode of Chinese landscape painting and figure painting. However, there are certain characteristics of their aesthetics, which are mainly reflected in the following aspects: First, there is no significant difference between the individual’s aesthetic processing of Chinese landscape painting and figure painting. Whether Chinese landscape painting or figure painting, the individual is processing more from the perspective of semantic representation. Second, the individuals’ processing of Chinese painting is automatic processing from the perspective of semantic representation and emotional awareness, and aesthetic processing from the emotional awareness may be related to the length of viewing time. As a vital component of Chinese traditional art and culture, aesthetic processing in Chinese landscape painting or figure painting is produced by the interaction of semantic representation and emotional awareness.

## Figures and Tables

**Table 1:** Average number of different types of words generated by different types of Chinese painting works.

	Content Words (Times)	Emotional Association Words (Times)
	*M* ± *SD*	*M* ± *SD*
Landscape paintings	147.45 ± 23.88	32.14 ± 9.52
Figure paintings	101.64 ± 16.53	75.26 ± 19.85

**Table 2:** Chi-square test results for different types of vocabulary generated by different types of Chinese painting works.

Types of Paintings	Types of Words	Frequency	Chi-Square	*df*	*p*
		*N*			
Landscape paintings	Content words	1407	731.74	1	0.00
	Emotional association words	292			
Figure paintings	Content words	979	44.23	1	0.00
	Emotional association words	706			

**Table 3:** Seven categorized test blocks of the IAT program.

Block	Character Description	Type of Stimulation	Key Response
1	Painting Classification	Landscape Paintings	F
		Figure Paintings	J
2	Attribute Word Classification	Objective Words	F
		Subjective Words	J
3	Compatible Joint Task 1	Landscape Paintings and Objective Words	F
		Figure Paintings and Subjective Words	J
4	Compatible Joint Task 2	Landscape Paintings and Objective Words	F
		Figure Paintings and Subjective Words	J
5	Attribute Word Classification	Subjective Words	F
		Objective Words	J
6	Incompatible Joint Task 1	Landscape Paintings and Subjective Words	F
		Figure Paintings and Objective Words	J
7	Incompatible Joint Task 2	Landscape Paintings and Subjective Words	F
		Figure Paintings and Objective Words	J

**Table 4:** IAT response time (ms) and effect values (logarithmic values).

	Compatible Joint Task	Incompatible Joint Task	IAT Effect Value
	*M* ± *SD*	*M* ± *SD*	*M* ± *SD*
Response time	723.35 ± 329.84	709.17 ± 330.75	−14.17 ± 440.83
Pair values	2.83 ± 0.15	2.82 ± 0.16	2.08 ± 0.62

## Data Availability

The datasets generated during and/or analyzed during the current study are not publicly available as the data are part of ongoing research. The data supporting the findings of this study are available from the corresponding author upon request.
